# The relationship between blood lipids and plasma amyloid beta is depend on blood pressure: a population-based cross-sectional study

**DOI:** 10.1186/s12944-020-1191-4

**Published:** 2020-01-14

**Authors:** Ningwei Hu, Ling Gao, Yu Jiang, Shan Wei, Suhang Shang, Chen Chen, Liangjun Dang, Jin Wang, Kang Huo, Meiying Deng, Jingyi Wang, Qiumin Qu

**Affiliations:** 1 Department of Neurology, The First Affiliated Hospital of Xi’an Jiaotong University, 277 West Yanta Rd, Xi’an, 710061 China; 2 Huyi Hospital of Traditional Chinese Medicine, Xi’an, China

**Keywords:** Alzheimer’s disease, Amyloid beta, Blood lipids, Blood pressure

## Abstract

**Background:**

It is believed that deposition of amyloid beta (Aβ) in the brain is the central pathological changes of Alzheimer’s disease (AD), which triggers a series of pathological processes. However, the relationship between dyslipidemia and AD is uncertain. Considering the peripheral Aβ levels are related to brain Aβ deposition, we explore the relationships between blood lipids and plasma Aβ.

**Methods:**

Participants who lived in the selected village of Xi’an for more than 3 years were enrolled, aged 40–85 years (*n* = 1282, 37.9% male). Fasting blood lipid, plasma Aβ levels, basic information and living habits were measured. Multiple linear regressions were used.

**Results:**

In total population, blood lipids were not associated with plasma Aβ. After stratified by blood pressure, serum total cholesterol (TC) and low-density lipoprotein (LDL-c) were positively associated with plasma Aβ_42_ levels (β_TC_ = 0.666, *P*_TC_ = 0.024; β_LDL-c_ = 0.743, *P*_LDL-c_ = 0.011, respectively) in normal blood pressure. LDL-c was negatively associated with plasma Aβ_40_ levels (β = − 0.986, *P* = 0.037) in high blood pressure.

**Conclusion:**

Elevated plasma Aβ_42_ levels are associated with higher TC and LDL-c in normal blood pressure. Elevated plasma Aβ_40_ levels are associated with lower LDL-c in high blood pressure. This indicated that the relationships between blood lipids and plasma Aβ were confounded by blood pressure.

## Introduction

Alzheimer’s disease (AD) is the most common cause of dementia. Toxic plaque formed by the deposition of amyloid beta (Aβ) peptide in the brain is the main characteristic pathogenesis of AD [1]. Aβ peptides are natural products of metabolism consisting of 39 to 43 amino acids, formed by fragmentation of amyloid-β protein precursor (APP) through the sequential enzymatic actions of secretases [2]. In the process of Aβ formation, APP is first cleaved by β-secretase (known as BACE1), releasing sAPPβ into the extra cellular fluid and cerebrospinal fluid (CSF). The remaining fragment is then cleaved by γ-secretase to produce damaging amyloid-β42 (Aβ_42_) and other Aβ isoform (Aβ_40_ down to Aβ_17_). In addition, full-length APP is also cleaved by β and α-secreatase to form Aβ_16_ down to Aβ_13_ [3]. Under normal circumstance, most of the production is amyloid-β40 (Aβ_40_) and only a small amount of Aβ_42_ which is more likely to deposit and has neurotoxic. Aβ present in the brain can be eliminated by various means, including degradation of Aβ degrading enzymes, cell clearance, blood brain barrier (BBB) transport, CSF and interstitial lymphatic drainage, clearance of peripheral cells and tissues, etc. Due to the imbalance of Aβ production and clearance, a large number of neuritic plaques (formed by Aβ deposition) are present in the cerebral cortex, hippocampus, and some subcortical nuclei. Aβ deposition in the brain may be the initiating factor in AD process, which is called “amyloid hypothesis” [4]. It has been suggested that Aβ levels in the brain and plasma are in a dynamic balance. Deposition of Aβ in the brain subsequently affects plasma concentration [5]. Peripheral transport of Aβ can reduce its accumulation in the brain, suggesting that the Aβ concentration in plasma is related to the deposition of Aβ in the brain [6].

Dyslipidemia is one of the important risk factor for cardiovascular disease and stroke. Numerous studies showed that blood lipids were also significantly associated with the risk of AD [7–9], but with conflicting results. Several epidemiological, laboratory research and clinical studies supported the hypothesis that higher levels of cholesterol may induce the development of AD [10–13], while others had not confirmed or inversed association with the risk of AD [14–16]. The effects of blood lipids levels on Aβ deposition in the brain were unclear.

Considering that plasma Aβ concentration is related to cerebral Aβ levels and mounting evidence had indicated that blood pressure is related to plasma Aβ levels significantly, we conducted a cross-sectional study to explore the effects of blood pressure on the relationships between blood lipids and plasma Aβ levels in a community population.

## Methods

### Participants

**Fig. 1:**
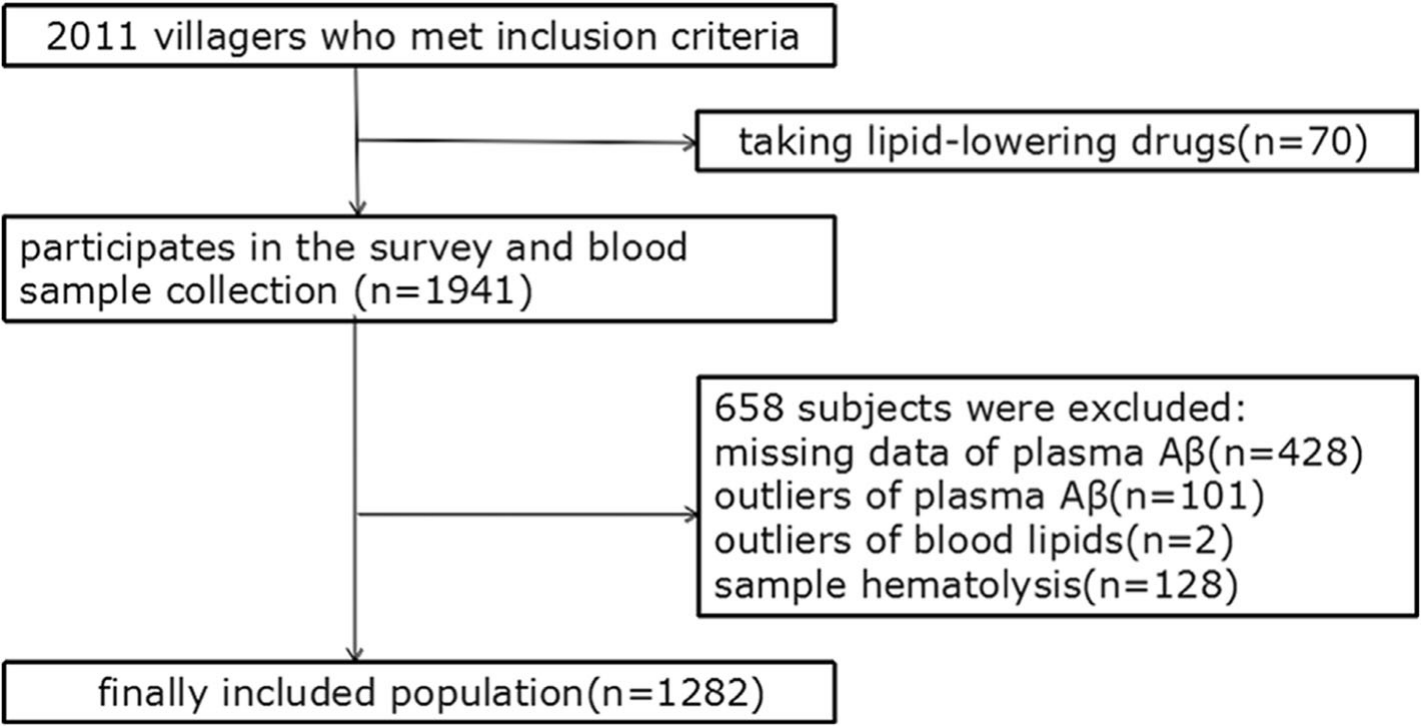
Flow chart of participant screening. Aβ, amyloid beta

From October 2014 to March 2015, all aged 40 or more villagers in Qubao village which taken by cluster sampling method near Xi’an were enrolled. There were similar lifestyles and population composition between this village and other rural areas of Xi’an. Inclusion criteria: 1) resident villager or who has lived in this area for 3 years or more, 2) agree to participate in this study and provided informed consent (*N* = 2011). Exclusion criteria: 1) severe cardiac, pulmonary, liver, kidney dysfunction, hematological, acute infection, or tumors, 2) those who have taken lipid-lowering drugs (*n* = 70), 3) those who showed aberrant plasma Aβ_42_, Aβ_40_ levels (*n* = 529) or blood lipids levels (*n* = 2) (exceeding 3 standard deviations from the mean), 4) sample hematolysis (*n* = 128). Total of 1282 participants were included in our analysis **(**Fig. 1**)**.

### Definition of covariates

The diseases and related conditions involved in this study are defined according to the guidelines [17, 18] as follows: 1) A person with a current systolic blood pressure (SBP) ≥140 mmHg and/or diastolic blood pressure (DBP) ≥90 mmHg is defined as high blood pressure. On the contrary, it is defined as normal blood pressure. Blood pressure measured with taking antihypertensive drugs and/or a current SBP ≥ 140 mmHg and/or a DBP ≥90 mmHg is defined as a hypertensive patient. 2) High fasting blood glucose (FBG) was defined as at least 7 mmol/L. 3) According the guidelines for the prevention and treatment of dyslipidemia in Chinese adults (revised edition 2016), high serum total cholesterol (TC) was defined as at least 5.18 mmol/L, high triglyceride (TG) was defined as at least 1.70 mmol/L, high serum low-density lipoprotein (LDL-c) was defined as at least 3.37 mmol/L and low serum high-density lipoprotein (HDL-c) was defined as less than 1.04 mmol/L. Any abnormality in blood lipids is defined as dyslipidemia. 4) Apolipoprotein E (ApoE ε4) carriers were defined as having at least one allele of ε4, while non-carriers referred to the ones without any ε4 allele.

### Questionnaire survey

A uniform questionnaire was used for all subjects, first of all, face to face consultation to collect general information, followed by physical examination and blood sample collection.

### Blood pressure measurement

Blood pressure (BP) was measured by a nurse using a mercury sphygmomanometer on each participant’s right arm with a regular adult cuff (Shanghai Medical Instruments Co. Shanghai, China) in the morning, before breakfast (from 8 a.m. to 10 a.m.). Setting BP was measure again after 10 min of rest, and the average of the twice was recorded.

### Laboratory evaluation

All subjects were collected 5 ml of venous blood sample from 8 a.m. in the morning (at least 8 h on an empty stomach). 1) Then the blood sample was placed in a serum tube containing a coagulant and was gently inverted upside and down and stood for 30 min. Next, the sample was centrifuged at 3000 rpm for 15 min at room temperature for 2 h and quickly stored at − 80 °C until later measurement. TC, TG, HDL-c and LDL-c levels were detected by enzymatic method using an automated biochemical analyzer (C501, Roche, Sweden). Quality indicators accord with the quality requirements set by the US National Cholesterol Education Program. 2) Plasma levels of Aβ_40_, Aβ_42_ were measured with commercially available quantitative enzyme-linked immunosorbent assay kits (ELISA, Yuanye Co. Shanghai, China), and the sensitivity of each assay was 1.0 pg/ml, respectively. Measurements were performed using an RT-6000 analyzer (Rayto Co. Shenzhen. China) at 450 nm, and concentrations were calculated from the standard curve. All measurements were performed in duplicate and the results averaged. The intra-assay and inter-assay coefficients of variation were less than 7 and 9%, respectively. 3) Determination of ApoE genotypes: According to manufacturer’s protocol, genomic DNA from blood samples in the EDTA anticoagulant tubes was extracted by blood genomic deoxyribonucleic acid (DNA) extraction kit (Tiangen Co. Beijing. China). We amplified 244 base pair of the ApoE gene fragment using a polymerase chain reaction (PCR) thermocycler, the length of which included two polymorphic sites at amino acid residues 112 and 158(15). Sequence of the PCR products was tested by Sanger sequencing (Sangon Co. Shanghai. China). Finally, we used direct interpretation of the sequencing chromatogram to determine the ApoE genotype.

### Statistical analysis

Before doing statistical analysis, we tested the distribution of each covariate by using Skewness, Kurtosis and P-P plots. Covariates that nearly conformed to normal distribution included age, pulse rate, waistline, hip circumference, BMI, SBP, DBP, TC, HDL-c, LDL-c which were expressed as mean (SD) and were compared between different groups by using an unpaired Student’s *t*-test. Non-normal distribution covariates included education, levels of TG and FBG were expressed as median (interquartile range) and were compared by Mann-Whitney U-test. Categorical variables were expressed as the number (percentage) and were compared by *X*^2^test. For serum lipid, participants were divided into dyslipidemia group and normal blood lipids group. Differences between two groups were compared. We used simple linear correlation scatter plot to roughly observe the linear trend between blood lipids and plasma Aβ levels. We performed multiple liner regression models to explore the statistical significance of the association after adjusting for other confounding factors including age, sex, education years, smoking, drinking, physical activity level, and history of heart disease, waist circumference, hip circumference, BMI, pulse rate, SBP, DBP, FBG and ApoE ε4 genotype (ApoE is involved in the regulation of Aβ metabolism, aggregation and deposition [19]).

Two secondary analyses were performed. First, associations between blood lipids and Aβ were analyzed in total study population. Because TC was collinear with LDL-c, two models were built. Model 1: TG, TC, HDL-c and other covariates mentioned above. Model 2: TG, LDL-c, HDL-c and other covariates mentioned above. Second, to rule out the possibility of BP affecting the relationship between blood lipids and plasma Aβ, we divided the total population into high blood pressure and normal blood pressure according to the standard of SBP greater than or equal to 140 mmHg and/or DBP greater than or equal to 90 mmHg and compared the relationship again in new two-tiered crowd (Model 3, Model 4).

All statistical analyses were performed with IBM SPSS Statistics version 24.0. A two-side *P* value of less than 0.05 was the threshold for statistical significance.

## Results

### Characteristics of the population

As shown in the Table 1, participants with dyslipidemia (*n* = 644) were older, higher ratios of hypertension, diabetes mellitus, higher levels of BMI, SBP, DBP, FBG, TC, LDL-c, TG and lower levels of education and HDL-c than those with normal lipids.

**Table 1:** Characteristics of the total study population

Characteristics	Total (*n* = 1282)	Dyslipidemia (*n* = 644)	Normal blood lipids (*n* = 638)	t or U or Chi square	df	*P* value
Age, years	55.70 (10.19)	56.74(10.1)	54.66(10.19)	−3.673	1280	<0.001
Male, n(%)	486(37.9)	242(37.6)	244(38.2)	0.061	1	0.806
Education, years	7(4,8)	7(3,8)	7(5,9)	190,461.5	–	0.023
Hypertension, n(%)	601(46.9)	356(55.3)	245(38.4)	36.663	1	<0.001
Diabetes mellitus, n(%)	146(11.4)	91(14.1)	55(8.6)	9.641	1	0.002
Cardiovascular disease, n(%)	73(5.7)	40(6.2)	33(5.2)	0.644	1	0.422
Transient ischemic attack, n(%)	23(1.8)	13(2.0)	10(1.6)	0.370	1	0.543
Stroke, n(%)	72(5.6)	41(6.4)	31(4.9)	1.374	1	0.241
Smoking, n(%)	349(27.2)	170(26.4)	179(28.1)	0.445	1	0.505
Drinking, n(%)	168(13.1)	93(14.4)	76(11.8)	2.030	1	0.154
Lack of physical activity, n(%)	225(17.6)	123(19.1)	102(16.0)	2.145	1	0.143
Pulse rate, bpm	75.48(8.74)	75.51(8.58)	75.44(8.90)	−0.151	1280	0.880
Waistline, cm	84.76(8.96)	86.82(8.99)	82.68(8.44)	−8.482	1280	<0.001
Hip circumference, cm	96.39(6.56)	97.48(6.76)	95.28(6.17)	−6.091	1280	<0.001
BMI, kg/m^2^	25.13(3.20)	25.69(3.32)	24.57(2.98)	−6.346	1297.91	<0.001
SBP, mmHg	132.16(19.08)	135.62(19.58)	128.67(17.92)	−6.637	1272.03	<0.001
DBP, mmHg	81.82(10.44)	83.63(10.83)	80.00(9.72)	−6.315	1267.75	<0.001
FBG, mmol/L	5.39(5.06, 5.77)	5.42(5.07, 5.91)	5.35(5.06, 5.69)	188,237	–	0.009
TG, mmol/L	1.43(1.03,1.99)	1.99(1.48, 2.47)	1.10(0.88, 1.39)	53,420	–	<0.001
TC, mmol/L	5.04(1.01)	5.57(1.02)	4.51(0.64)	−22.296	1086.03	<0.001
LDL-c, mmol/L	3.31(0.89)	3.78(0.89)	2.839(0.56)	−22.888	1085.95	<0.001
HDL-c, mmol/L	1.41(0.31)	1.37(0.32)	1.46(0.30)	5.168	1280	<0.001
ApoE ε4, n(%)	173(13.5)	92(14.3)	81(12.7)	0.180	2	0.667

Unpaired Student’s *t*-test and mean ± SD were used to compare the difference of the approximately normally distributed continuous variables between dyslipidemia and normal blood lipids. Mann-Whitney U test and mediam (quartile) were used for the skew distributional data and Chi square and percentage were used for categorical variables. Data are mean (SD), median (interquartile range), or number (percentage). BMI, body mass index. SBP, systolic blood pressure. DBP, diastolic blood pressure. FBG, fast blood glucose. TC, total cholesterol. TG, triglyceride. HDL-c, high-density lipoprotein. LDL-c, low-density lipoprotein. ApoE, apolipoprotein E

### Association between plasma Aβ levels and blood lipids in the total population

**Fig. 2:**
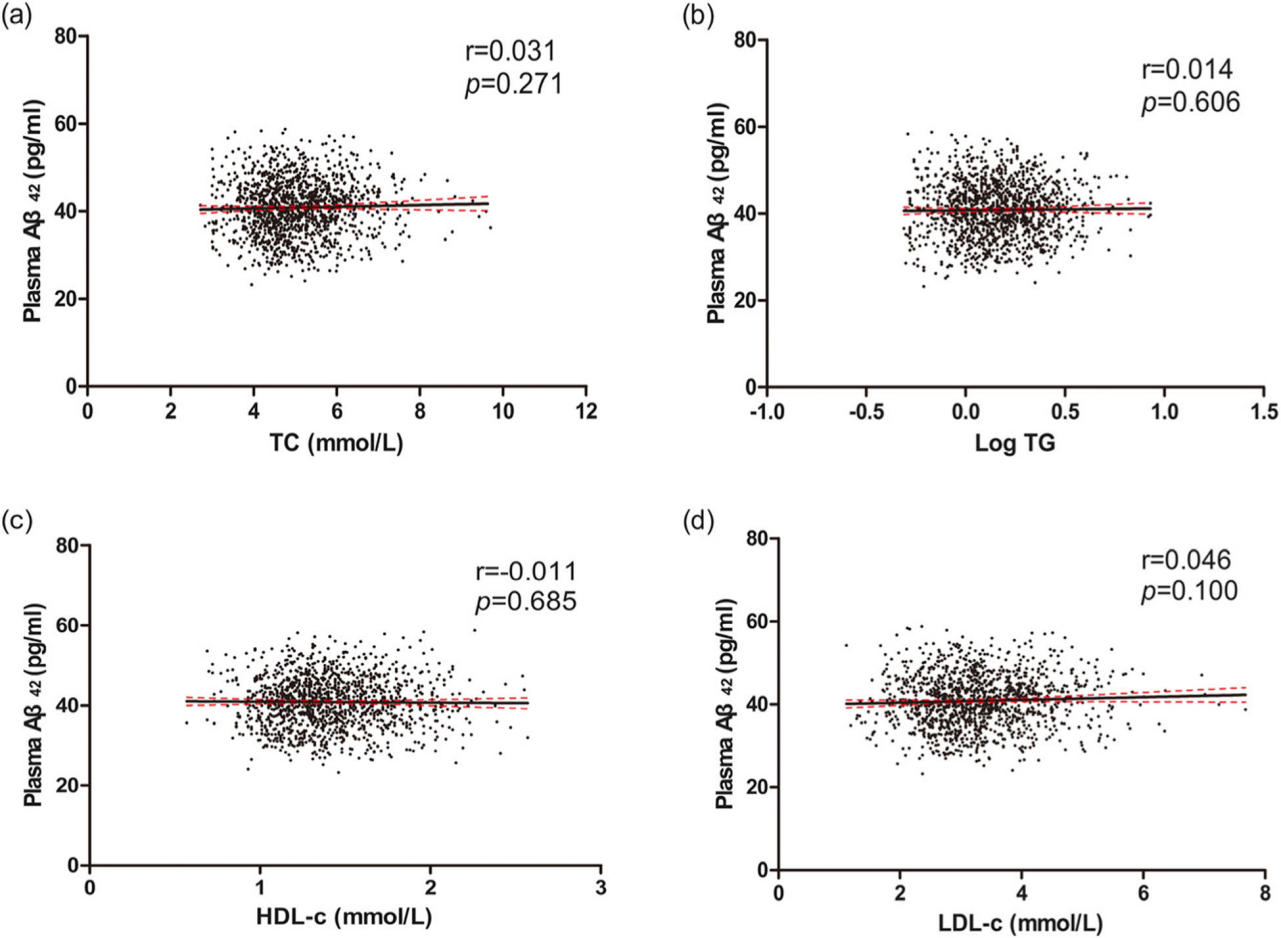
Correlations of TC, Log TG, HDL-c, LDL-c and plasma Aβ_42_ in total study population. Simple linear correlations between TC, Log TG, HDL-c, LDL-c and plasma Aβ_42_ were shown respectively in picture (**a, b, c, d**). Aβ, amyloid beta. TC, total cholesterol. TG, triglyceride. HDL-c, high-density lipoprotein. LDL-c, low-density lipoprotein

**Fig. 3:**
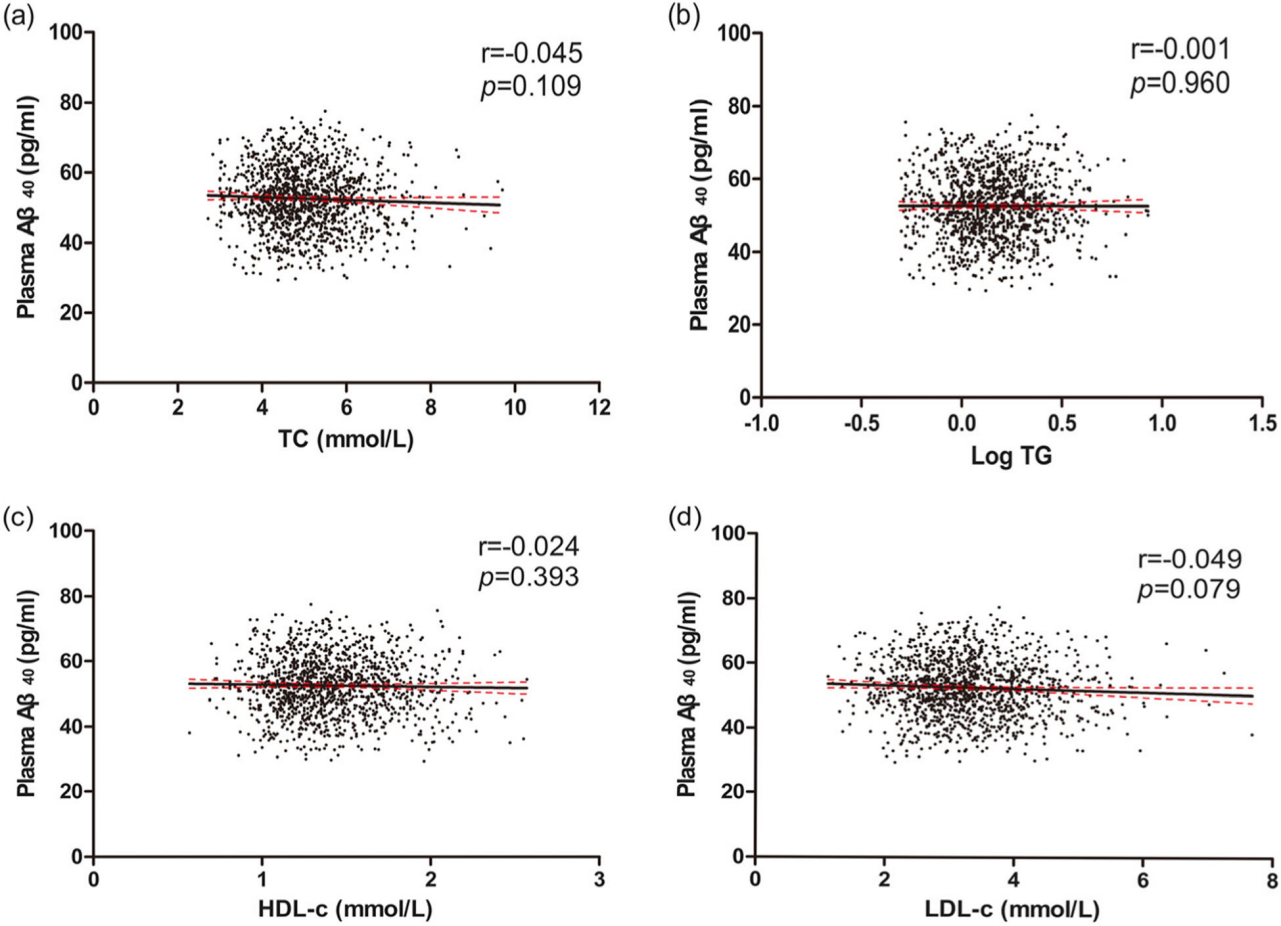
Correlations of TC, Log TG, HDL-c, LDL-c and plasma Aβ_40_ in total study population. Simple linear correlations between TC, Log TG, HDL-c, LDL-c and plasma Aβ_40_ were shown respectively in picture (**a, b, c, d**). Aβ, amyloid beta. TC, total cholesterol. TG, triglyceride. HDL-c, high-density lipoprotein. LDL-c, low-density lipoprotein

In the total population, plasma Aβ levels had no differences between dyslipidemia group and normal lipids group **(**Table 2**)**. No linear trends were found **(**Figs. 2, 3**)**. After adjusting for confounding factors as described above, no correlations were found between blood lipids and plasma Aβ levels **(**Table 3**)**.

**Table 2:** Comparison of plasma Aβ in Dyslipidemia group and Normal blood lipids in total study population (*n* = 1282)

	Aβ_42_(pg/ml)				Aβ_40_(pg/ml)			
	mean (SD)	t	df	*P*	mean (SD)	t	df	*P*
Dyslipidemia (n = 644)	40.97(6.11)	−0.889	1269.67	0.374	52.31(8.79)	0.865	1280	0.387
Normal blood lipids (n = 638)	40.65(6.62)				52.74(9.04)			
High TC (*n* = 515)	41.11(6.16)	−1.389	1142.34	0.165	52.20(8.96)	1.077	1280	0.282
Normal TC (*n* = 767)	40.61(6.50)				52.74(8.89)			
High TG (*n* = 447)	40.98(6.03)	−0.715	1280	0.475	52.65(8.91)	− 0.371	1280	0.710
Normal TG (*n* = 835)	40.72(6.54)				52.46(8.92)			
High LDL-c (*n* = 544)	41.10(6.03)	−1.398	122,239	0.162	52.09(8.96)	1.485	1280	0.138
Normal LDL-c (*n* = 738)	40.60(6.60)				52.84(8.88)			
Low HDL-c (*n* = 116)	40.89(6.48)	−0.142	1280	0.887	53.18(9.34)	−0.825	1280	0.409
Normal HDL-c (*n* = 1166)	40.80(6.36)				52.46(8.87)			

Unpaired Student’s *t* –test were used to compare the difference of plasma Aβ_42_, Aβ_40_ between the groups of the covariates. Data are shown as mean (SD). Aβ, amyloid beta. TC, total cholesterol. TG, triglyceride. HDL-c, high-density lipoprotein. LDL-c, low-density lipoprotein

**Table 3:** Multiple linear regression of blood lipids and plasma Aβ levels in total study participants (*n* = 1282)

	Aβ_42_(pg/ml)			Aβ_40_(pg/ml)		
	β	t	*P* value	β	t	*P* value
Model 1						
TG	−0.030	−0.122	0.903	0.087	0.256	0.798
TC	0.254	1.156	0.248	−0.356	−1.156	0.248
HDL-c	−0.448	−0.615	0.539	−0.625	− 0.611	0.541
ApoE ε4	0.955	1.813	0.070	0.278	0.376	0.707
Model 2						
TG	−0.001	−0.004	0.997	0.042	0.134	0.893
LDL-c	0.325	1.483	0.138	−0.442	−1.439	0.150
HDL-c	−0.252	−0.385	0.700	−0.908	− 0.988	0.323
ApoE ε4	0.946	1.797	0.073	0.288	0.390	0.697

β, the unstandardized regression coefficient

Model 1: adjust for sex, age, education years, smoking, drinking, lack of physical activity, cardiovascular disease, waistline and lip circumference, pulse rate, SBP, DBP, FBG, TC, TG, HDL-c, BMI and ApoE genotype. BMI, body mass index. SBP, systolic blood pressure. DBP, diastolic blood pressure. FBG, fast blood glucose Aβ, amyloid beta. TC, total cholesterol. TG, triglyceride. HDL-c, high-density lipoprotein. LDL-c, low-density lipoprotein. ApoE, apolipoprotein E

Model 2: adjust for sex, age, education years, smoking, drinking, lack of physical activity, cardiovascular disease, waist and lip circumference, pulse rate, SBP, DBP, FBG, LDL-c, TG, HDL-c, BMI and ApoE genotype. BMI, body mass index. SBP, systolic blood pressure. DBP, diastolic blood pressure. FBG, fast blood glucose Aβ, amyloid beta. TC, total cholesterol. TG, triglyceride. HDL-c, high-density lipoprotein. LDL-c, low-density lipoprotein. ApoE, apolipoprotein E

### The effects of blood pressure on plasma Aβ levels and blood lipids levels

After re-stratified by blood pressure **(**Table 4**)**, participants with high blood pressure (*n* = 548) were older, more diabetes mellitus, cardiovascular disease and stroke, high levels of waistline, hip circumference, BMI, SBP, DBP, FBG, TC, LDL-c, TG and a lower levels of education and HDL-c levels.

**Table 4:** Comparisons of High blood pressure group and Normal blood pressure group

Characteristics	High blood pressure group (*n* = 548)	Normal blood pressure group (*n* = 734)	t or U or Chi square	df	*P* value
Age, years	59.03 (9.88)	53.21 (9.70)	−10.536	1280	<0.001
Male, n(%)	210 (38.3)	276(37.6)	0.069	1	0.793
Education, years	6 (3,8)	8 (5,9)	171,805	–	<0.001
Diabetes mellitus, n(%)	88(16.1)	58 (7.9)	20.684	1	<0.001
Cardiovascular disease, n(%)	40 (7.3)	33 (4.5)	4.592	1	0.032
Transient ischemic attack, n(%)	12 (2.2)	11 (1.5)	0.851	1	0.356
Stroke, n(%)	46 (8.4)	26 (3.5)	13.934	1	<0.001
Smoking, n(%)	145(26.5)	204 (27.8)	0.281	1	0.596
Drinking, n(%)	74 (13.5)	94 (12.8)	0.134	1	0.714
Lack of physical activity, n(%)	112 (20.4)	113 (15.4)	5.514	1	0.019
Pulse rate, bpm	76.35 (9.11)	74.82 (8.40)	−3.115	1280	0.002
Waistline, cm	86.77 (9.21)	83.26 (8.47)	−6.979	1121.85	<0.001
Hip circumference, cm	97.41(6.82)	95.62 (6.25)	−4.867	1280	<0.001
BMI, kg/m^2^	25.87(3.37)	24.58 (2.95)	−7.177	1087.29	<0.001
SBP, mmHg	149.26 (14.88)	119.39 (9.45)	−41.205	867.62	<0.001
DBP, mmHg	89.80 (9.65)	75.87 (6.17)	−29.572	871.13	<0.001
FBG, mmol/L	5.48 (5.15,6.01)	5.32 (5.01,5.66)	163,777	–	<0.001
TG, mmol/L	1.64(1.17,2.22)	1.28 (0.97,1.78)	150,027	–	<0.001
TC, mmol/L	5.14 (1.00)	4.96 (1.00)	−3.177	1280	0.002
LDL-c, mmol/L	3.40 (0.88)	3.24 (0.88)	−3.182	1280	<0.001
HDL-c, mmol/L	1.39 (0.31)	1.43 (0.32)	1.893	1280	0.059
ApoE ε4, n(%)	70 (14.06)	103 (15.42)	0.426	2	0.808
Aβ_42_, pg/mL	40.64 (6.32)	40.94 (6.41)	0.842	1280	0.400
Aβ_40_, pg/mL	53.05(8.82)	52.13(8.98)	−1.828	1280	0.068

Unpaired Student’s *t*-test and mean ± SD were used to compare the difference of the approximately normally distributed continuous variables between high blood pressure and normal blood pressure group. Mann-Whitney U test and mediam (quartile) were used for the skew distributional data and Chi square and percentage were used for categorical variables. Data are mean (SD), median (interquartile range), or number (percentage). BMI, body mass index. SBP, systolic blood pressure. DBP, diastolic blood pressure. FBG, fast blood glucose. Aβ, amyloid beta. TC, total cholesterol. TG, triglyceride. HDL-c, high-density lipoprotein. LDL-c, low-density lipoprotein. ApoE, apolipoprotein E

### Association of plasma Aβ levels and blood lipids stratified by blood pressure

**Fig. 4:**
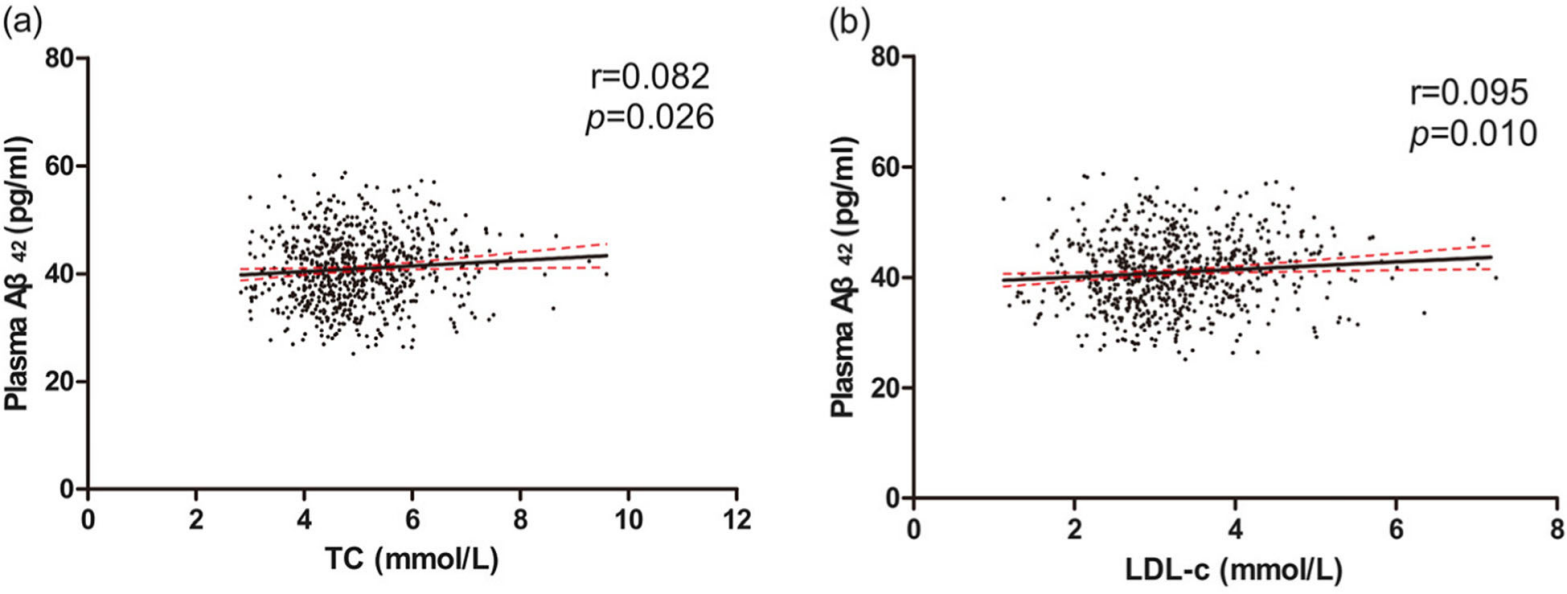
Correlations of TC, LDL-c and plasma Aβ_42_ in normal blood pressure group. Simple linear correlations between TC, LDL-c and plasma Aβ_42_ were shown respectively in picture (**a, b**). Aβ, amyloid beta. TC, total cholesterol. LDL-c, low-density lipoprotein

In normal blood pressure group, Aβ_42_ levels were higher in the high TC and high LDL-c group than that in the normal group **(**Table 5**)**. Positive linear trends were found between TC, LDL-c levels and plasma Aβ_42_ levels in normal blood pressure group **(**Fig. 4a, b**)**. Negative linear trend was found between LDL-c levels and plasma Aβ_40_ levels in high blood pressure group (r = − 0.089, *P* = 0.038). Consistent with previous analysis, TC and LDL-c were independently and positively associated with plasma Aβ_42_ levels after re-stratified in the normal blood pressure. LDL-c was negatively associated with plasma Aβ_40_ levels in high blood pressure group **(**Table 6**)**.

**Table 5:** Comparison of plasma Aβ between Dyslipidemia group and Normal blood lipids group stratified by blood pressure

	High blood pressure group (*n* = 548)								Normal blood pressure group (*n* = 734)							
	Aβ_42_(pg/ml)				Aβ_40_(pg/ml)				Aβ_42_(pg/ml)				Aβ_40_(pg/ml)			
	mean (SD)	t	df	*P*	mean (SD)	t	df	*P*	mean (SD)	t	df	*P*	mean (SD)	t	df	*P*
Dyslipidemia (*n* = 644)	40.62(6.09)	0.095	546	0.964	53.02(8.82)	0.107	546	0.458	41.32(6.11)	−1.432	713.43	0.145	51.61(8.72)	1.403	732	0.042
Normal blood lipids (*n* = 638)	40.67(6.63)				53.10(8.83)				40.64(6.62)				52.54(9.16)			
High TC (*n* = 515)	40.60(6.01)	0.113	546	0.910	52.81(8.98)	0.585	546	0.559	41.57(6.26)	−2.028	732	0.043	51.64(8.92)	1.120	732	0.263
Normal TC (*n* = 767)	40.67(6.56)				53.25(8.69)				40.58(6.47)				52.41(9.00)			
High TG (*n* = 447)	40.85(5.93)	−0.708	546	0.479	53.24(8.88)	−0.469	546	0.640	41.16(6.17)	−0.560	732	0.576	51.90(8.92)	0.416	732	0.677
Normal TG (*n* = 835)	40.46(6.62)				52.89(8.78)				40.86(6.50)				52.21(9.00)			
High LDL-c (*n* = 544)	40.53(5.99)	0.377	544.65	0.706	52.55(9.09)	1.240	546	0.215	41.59(6.04)	−2.281	655.74	0.023	51.70(8.84)	1.063	732	0.288
Normal LDL-c (*n* = 738)	40.73(6.60)				53.48(8.57)				40.51(6.61)				52.42(9.06)			
Low HDL-c (*n* = 116)	42.00(6.85)	−1.663	546	0.097	53.54(8.09)	−0.429	546	0.668	39.95(6.04)	1.281	732	0.201	52.87(10.34)	−0.596	70.78	0.553
Normal HDL-c (*n* = 1166)	40.49(6.25)				53.00(8.90)				41.03(6.44)				52.06(8.84)			

Unpaired Student’s t –test were used to compare the difference of plasma Aβ_42_, Aβ_40_ between the groups of the covariates. Data are shown as mean (SD) Aβ, amyloid beta. TC, total cholesterol. TG, triglyceride. HDL-c, high-density lipoprotein. LDL-c, low-density lipoprotein

**Table 6:** Multiple linear regression models of blood lipids and plasma Aβ levels stratified by blood pressure

	High blood pressure group (*n* = 548)						Normal blood pressure group (*n* = 734)					
	Aβ_42_(pg/ml)			Aβ_40_(pg/ml)			Aβ_42_(pg/ml)			Aβ_40_(pg/ml)		
	β	t	*P*	β	t	*P*	β	t	*P*	β	t	*P*
Model 3												
TG	0.661	1.849	0.065	0.618	1.220	0.223	−0.532	−1.609	0.108	−0.224	−0.480	0.631
TC	− 0.442	− 1.273	0.203	− 0.885	− 1.886	0.060	0.666	2.267	0.024	0.084	0.204	0.838
HDL-c	−1.525	− 1.392	0.165	0.503	0.324	0.746	0.100	0.102	0.919	−1.566	−1.130	0.259
ApoE ε4	0.305	0.372	0.710	−0.727	−0.627	0.531	1.207	1.755	0.080	1.083	1.117	0.264
Model 4												
TG	0.552	1.685	0.093	0.466	1.006	0.315	−0.423	−1.371	0.171	−0.185	− 0.426	0.670
LDL-c	−0.363	−1.088	0.277	−0.986	−2.090	0.037	0.743	2.558	0.011	0.013	0.031	0.976
HDL-c	−1.942	−1.935	0.053	−0.259	−0.182	0.855	0.689	0.786	0.432	−1.435	−1.160	0.247
ApoE ε4	0.304	0.370	0.711	−0.707	−0.610	0.542	1.203	1.751	0.080	1.093	1.128	0.260

β, the unstandardized regression coefficient

Model 3: adjust for sex, age, education years, smoking, drinking, lack of physical activity, cardiovascular disease, waistline and lip circumference, pulse rate, SBP, DBP, FBG, TC, TG, HDL-c, BMI and ApoE genotype. BMI, body mass index. SBP, systolic blood pressure. DBP, diastolic blood pressure. FBG, fast blood glucose Aβ, amyloid beta. TC, total cholesterol. TG, triglyceride. HDL-c, high-density lipoprotein. LDL-c, low-density lipoprotein. ApoE, apolipoprotein E

Model 4: adjust for sex, age, education years, smoking, drinking, lack of physical activity, cardiovascular disease, waist and lip circumference, pulse rate, SBP, DBP, FGB, LDL-c, TG, HDL-c, BMI and ApoE genotype. BMI, body mass index. SBP, systolic blood pressure. DBP, diastolic blood pressure. FBG, fast blood glucose Aβ, amyloid beta. TC, total cholesterol. TG, triglyceride. HDL-c, high-density lipoprotein. LDL-c, low-density lipoprotein. ApoE, apolipoprotein E

## Discussion

In this population-based study, we found that TC and LDL-c were positively correlated with plasma Aβ_42_ levels in normal blood pressure. LDL-c was negatively associated with plasma Aβ_40_ levels in the high blood pressure. This finding was independent of age, sex, ApoE ε4 and other confounding factors. This indicated that the relationships between blood lipids and plasma Aβ were confounded by blood pressure.

Studies have shown that hyperlipidemia may play a role in the development of AD [20]. A 13-year follow-up study showed that higher LDL-c and TC levels were associated with an increased risk of AD [7]. A study containing 7053 community-dwelling elderly suggested low TG was associated with decreased incident AD in women [21]. Elevated HDL-c levels might be associated with a decreased risk of AD were also found in elderly individuals [8]. Therefore, elevated blood lipids may play a role in the progression of AD [9].

However, the relationship between blood lipids and plasma Aβ is not fully determined. Positively correlation of HDL-c levels with Aβ_42_ in not using statins was observed and no relationships of Aβ_42_ with TC and LDL-c [22]. Inverse trend was observed between HDL-c and Aβ_42_ levels, although not significant [23]. Regression analysis considering the multiple influences of baseline parameters TC, LDL-c, HDL-c, TG, BMI, lnHbA1c and presence of at least one ApoE ε4 allele on the lnAβ_42_ at 5 years revealed TC as the only significant predictor. Excluding TC from the list of independent variables, LDL-c was the single negative predictor [24]. A double-blind, randomized, placebo-controlled study showed that after giving subjects with lovastatin 40 mg or 60 mg per day for 3 months, serum Aβ concentrations were lower than baseline measurements compared with the placebo group [25]. There was also reports in the literature that pravastatin at 10 mg/d does not decrease plasma levels of either Aβ_40_ or Aβ_42_ in humans [26]. The possible reason for the difference between the reported results in the literature and our study may be the research population, inclusion criteria, exclusion criteria and method for measuring Aβ has not been unified [27]. Compared with the INNO-BIA assay, the ELISA measured Aβ_40_ levels are slightly lower and Aβ_42_ levels are slightly higher [27]. Our previous research has proved the methods are credible [28, 29]. More research is still needed.

In present study, we did not find the relationship between dyslipidemia and plasma Aβ levels in the total population. However, after stratified by blood pressure, we found that TC and LDL-c were positively correlated with plasma Aβ_42_ levels in normal blood pressure, but not in the hypertension group, indicated that the relationship between blood lipids and Aβ is confounded by the blood pressure. Hypertension is the most important risk factor for cardiovascular disease and stroke. Also, growing evidence indicate that hypertension is a major risk factor for AD. Hypertension has an effect on blood lipids and Aβ [30]. Elevated blood pressure had effects on the Aβ [31–33]. Our previous study found that elevation in PP was associated with increased plasma Aβ_40_ and decreased log-transformed soluble advanced glycosylation end product-specific receptor (sRAGE), the underlying mechanism may be relevant to peripheral Aβ clearance [31]. Therefore, we explored the effect of blood pressure on the relationship between blood lipids and plasma Aβ.

The mechanism of dislipidemia related to plasma Aβ levels is not clear. Hypercholesterolemia may cause the deposition of Aβ in the brain by affecting the translocation of endothelial cells across the BBB [34]. The injured BBB in turn induces inflammation, resulting in an increase gap of brain microvascular endothelial cells [35, 36]. The damage of BBB may affect the expression of low-density lipoprotein receptor-related protein 1 (LRP1) and decrease the function of Aβ transport out of the brain. It may also promotes the expression of RAGE and increase the transport of plasma Aβ to the central nervous system, which ultimately causes Aβ deposition in the brain [37]. These findings suggest that dyslipidemia is associated with increased Aβ deposition in the brain. The process of brain Aβ from the center to the periphery is its main pathway of clearance, elevated plasma Aβ levels associated with increased Aβ deposition in the brain [38]. Therefore, blood lipids are associated with plasma Aβ may relate to the increased deposition of Aβ in the brain.

Recent years, study had also suggested that hyperlipidemia can affect Aβ metabolism [39]. TC is mainly concentrated in membrane microdomains termed lipid rafts where considerable evidence indicates that the amyloidogenic processing of APP largely occurs [39]. TC can enhance the activity of BACE1 (the rate-limiting enzyme for Aβ generation) and promote it’s localization to lipid rafts, otherwise, it can also act as a positive regulator of γ-secretase to further increase the activity of it [40]. BACE1 transcription increased in mice feed with high-fat and TC, suggesting that hypercholesterolemia increases the production of Aβ by affecting the activity of secretase [41]. In addition, increased TC in cell membranes can inhibit the function of α-secretase, promote the cleavage of APP by β-secretase and γ-secretase, and eventually lead to increased Aβ production [42].

An important question is why TC and LDL-c are related to plasma Aβ_42_ in normal blood pressure, and why LDL-c is correlated with plasma Aβ_40_ in high blood pressure rather than Aβ_42_. Aβ peptides are mainly produced in the brain, are transported to the cerebrospinal fluid and plasma, and are degraded in the periphery [43]. This degradation is of importance as it allows lowering the whole brain Aβ content. Aβ peptides, particularly Aβ_42_, are highly water insoluble molecules requiring lipid environments to be transported to the places of their degradation or excretion [44]. In addition, in normal blood pressure, the blood vessels walls are not damaged and transportation of Aβ is unrestricted. This is not the case in high blood pressure, where blood vessels might be damaged and transportation of Aβ peptides is consequently deteriorated. Therefore, in normal blood pressure, Aβ_42_ correlated with TC and LDL-c may as it’s strongly hydrophobic and the integrity of the vascular wall. In high blood pressure, Aβ_40_ peptides negatively associated with LDL-c as lower amount of Aβ peptides are transported through the blood vessels at all. Moreover, Aβ is highly hydrophobic peptides and requires lipid environment for its solubility. Positive correlation of TC and LDL-c with Aβ_42_ might simply reflect the better condition for solubility.

### Limitations

First, the design of this study did not allow for causal assumptions between plasma Aβ levels and dyslipidemia. It was difficult to determine whether dyslipidemia led to plasma Aβ change. The results need to be validated in additional longitudinal cohort studies. Second, we did not analyze the relationships of dyslipidemia and plasma Aβ levels in mild cognitive impairment (MCI) or dementia patients because of the rather small sample size. Third, Aβ deposition in the brain or CSF could not be obtained. The effects of peripheral Aβ clearance on brain Aβ accumulation must be investigated. Finally, we did not detect blood oxidized low-density lipoprotein (ox-LDL) level. It has been reported that ox-LDL is more toxic and plays a more important role in the pathogenesis of AD [45].

## Conclusions

In summary, out research find that elevated plasma Aβ_42_ levels are associated with higher TC and higher LDL-c in normal blood pressure. Elevated plasma Aβ_40_ levels are associated with lower LDL-c in high blood pressure. This indicated that the relationship between blood lipids and plasma Aβ was confounded by blood pressure. Considering the close relationship between plasma Aβ and deposition in the brain, we explore the relationship between plasma Aβ and blood lipids to provide some help for the auxiliary diagnosis of AD. Additional large-scale cohort studies and convincible evidence-based medical researches are required.

## Data Availability

The data used in this study are available from the corresponding author if needed.

## Abbreviations

AD: Alzheimer’s disease
APP: Amyloid beta protein precursor
Aβ: Amyloid beta
BBB: Blood brain barrier
DBP: Diastolic blood Pressure
FBG: Fast blood glucose
HDL-c: High-density lipoprotein
LDL-c: Low-density lipoprotein
LRP1: Low-density lipoprotein receptor-related protein 1
PCR: Polymerase chain reaction
PP: Pulse pressure
RAGE: Advanced glycosylation end product-specific receptor
SBP: Systolic blood pressure
TC: Total cholesterol
TG: Triglyceride
